# Co-segregation of the c.489+3A>G variant with p.Cys1400Ter pathogenic *CFTR* mutation in Cyprus: prevalence and clinical implications

**DOI:** 10.1186/s13023-025-03714-3

**Published:** 2025-04-29

**Authors:** Panayiotis K. Yiallouros, Pinelopi Anagnostopoulou, Panayiotis Kouis, Andreas Μ. Matthaiou, Tonia Adamidi, Phivos Ioannou, George Christopoulos, Constantina Costi, Leonidas A. Phylactou, Pavlos Fanis, Vassos Neocleous

**Affiliations:** 1 Respiratory Physiology Laboratory, Medical School, University of Cyprus, Nicosia, Cyprus; 2 Paediatric Pulmonology Unit, ‘Archbishop Makarios III’ Hospital, Nicosia, Cyprus; 3 Pulmonology Clinic, Nicosia General Hospital, Nicosia, Cyprus; 4 Molecular Genetics Thalassaemia Department, The Cyprus Institute of Neurology and Genetics, Nicosia, Cyprus; 5 Department of Molecular Genetics, Function and Therapy, The Cyprus Institute of Neurology and Genetics, Nicosia, Cyprus

**Keywords:** *CFTR* gene, c.489+3A>G variant, p.Cys1400Ter, Co-segregation, Cystic fibrosis, CFTR-related disorder, Patient registry

## Abstract

**Background:**

The high variety of mutations found in the Cystic Fibrosis Transmembrane Regulator (*CFTR*) gene is responsible for the clinical heterogeneity observed in people with Cystic Fibrosis (CF) and the atypical manifestations in CFTR-related disorders (CFTR-RD). The intronic c.489+3A>G (c.621+3A>G) variant has been reported to have questionable pathogenicity, although its alleged severity was probably due to its co-segregation *in cis* with another undetected mutation, as previously reported from countries in the Mediterranean region. In the island of Cyprus, several rare *CFTR* variants have been previously identified, among them the c.489+3A>G in co-segregation with the pathogenic p.Cys1400Ter (cDNA name = c.4200_4201del or legacy name = 4332delTG) mutation. We aimed to investigate the prevalence of these variants in Cyprus and describe their clinical impact in patients and carriers.

**Results:**

The intronic variant c.489+3A>G has been so far identified to co-segregate with the pathogenic p.Cys1400Ter mutation in the same allele in six unrelated Cypriot families and in total of 20 subjects. Three of them were diagnosed with CF, presenting with persistent respiratory symptoms, pancreatic insufficiency and a second CF-causing mutation. Two were diagnosed with CFTR-RD, presenting with bronchiectasis, intermediate sweat test and a second mutation known to cause CFTR-RD. Also, four carriers had a high suspicion of CFTR-RD, with bronchiectasis or emphysema and intermediate sweat test, although due to the lack of another *CFTR* mutation and a second functional test, definite diagnosis has not been made. Haplotype analysis provided evidence of a common haplotype in all individuals with co-segregation of the c.489+3A>G variant with p.Cys1400Ter mutation.

**Conclusion:**

The intronic c.489+3A>G variant co-segregates extensively with p.Cys1400Ter in Cyprus as an ancestral combination due to a possible founder effect. Before providing genetic counselling to subjects identified through population screening to harbour the c.489+3A>G variant, extensive analysis of *CFTR* including gene rearrangements should be performed to identify possible other mutations *in cis*, especially in Mediterranean countries where this complex allele is probably common. Further research is warranted to fully delineate the clinical implications of the *in cis* co-segregation of p.Cys1400Ter with c.489+3A>G, even in the absence of pathogenic variants in the other *CFTR* allele.

## Introduction

More than 2000 pathogenic mutations have been reported to date in Cystic Fibrosis Transmembrane Regulator (CFTR) gene and this explains, at least partly, the clinical heterogeneity observed in people with Cystic Fibrosis (pwCF) and the atypical manifestations in CFTR-related disorders (CFTR-RD) [1]. The most common pathogenic mutation across the world is p.Phe508del, but in Southern European populations the proportion of non p.Phe508del alleles in CF carriers is high [2, 3], and not surprisingly the pathogenicity of some of these variants is disputed. Defining the exact role of these nucleotide substitutions is essential to provide genetic counselling to subjects identified through population screening.

The intronic c.489+3A>G (c.621+3A>G) variant was first reported to the Cystic Fibrosis Mutation Database (CFTR1) [4] as a neutral polymorphism. According to the CFTR2 database that superseded CFTR1, the c.489+3A>G variant has varying consequences [1]. In some cases, individuals with this variant, in combination with another CF-causing variant, may develop CF. However, in other instances, individuals with the same combination of variants may not exhibit any symptoms of CF. Subsequently, this variant was identified in four Greek pwCF as a severe disease-causing mutation, disrupting the splicing of exon 4 and leading to the formation of a non-functional CFTR protein [5]. Recent functional and clinical evidence from Italy suggested that the c.489+3A>G variant should not be classified as a CF disease-causing mutation [6]. Another Italian study [7] reported a patient with CFTR-RD who harboured the c.489+3A>G/p.Phe508del *in trans,* exhibited a sweat chloride level of 40 mmol/L, and presented with isolated bronchiectasis. These studies implied that the severe phenotype reported by Tzeti et al. in four Greek pwCF carrying c.489+3A>G was likely due to an undetected *in cis* mutation that was not identified by the mutation analysis protocol performed at the time [6]. Interestingly, complex alleles involving c.489+3A>G and p.Cys1400Ter *in cis* have been identified recently in a CF patient from Algeria [8] and another from France [9]. Of the two co-segregated mutations in these patients, p.Cys1400Ter (cDNA name = c.4200_4201 delor legacy name = 4332 delTG) is known to be pathogenic and its frequency is extremely low (0.00013 in 18/142,036), while c.489+3A>G variant’s frequency is even lower (0.00007 in 10/142,036) [1].

A CF registry was established in Cyprus in 2017. This was created using the operating procedures of the European Cystic Fibrosis Society Patient Registry (ECFSPR) [10]. The p.Phe508del mutation accounts for approximately 45% of CF-causing mutations in the country. Additionally, several rare genotypic profiles have been identified, including the unique p.Leu346Pro mutation, the intra-CFTR rearrangement known as CFTR-dup2, and a complex allele featuring both p.Cys1400Ter and c.489+3A>G mutations *in cis*. This complex pathogenic variant *in cis* was identified in three unrelated patients, representing 2.9% of the CFTR alleles in the country’s CF registry in 2019 [10]. Given that this combination has been previously reported in populations around the Mediterranean Sea [8, 9], we hypothesized that c.489+3A>G co-segregation with p.Cys1400Ter may represent an ancestral variant that spread in Cyprus due to a founder effect. In this study, we sought to investigate the prevalence of the c.489+3A>G and p.Cys1400Ter variants, both independently and in co-segregation within the Cypriot population. Furthermore, we aimed to characterize the clinical impact of these mutations in affected patients and carriers.

## Methods

### Patients’ and carriers’ identification

Over the past 4.5 years, from January 2020 to July 2024, we have conducted an active search for the specific *CFTR* variants, c.489+3A>G and p.Cys1400Ter. This search was initiated within the family pedigrees of the three known CF patients identified by 2019, as well in the families of any new carriers or patients identified during this 4.5-year timeframe.

The participants’ status was defined as (1) ***CF patient***, if they had (a) two sweat chloride test values of ≥ 60 mmol/L, or (b) one sweat chloride test value of ≥ 60 mmol/L and two disease-causing *CFTR* mutations, or (c) typical CF features at clinical presentation and two disease-causing CFTR mutations if sweat chloride test value was between 30 and 59 mmol/L or not reported [2]; (2) ***CFTR-related disorder***, if patients were bearing one CF mutation and one CFTR-related disorder mutation or two CFTR-related disorder mutations and a sweat chloride between 30 and 59 mmol/L [11]; and (3) ***Carrier***, if they had these variants on one allele, either alone or *in cis* and a sweat chloride ≤ 29 mmol/L.

Genotyping and measurement of sweat chloride with pilocarpine iontophoresis stimulation of a localized skin area was performed for all participants [12]. The earliest date when at least one of the above criteria was fulfilled was considered as the date of diagnosis. A total of seven (four males and three females) unrelated probands and eighteen other first-degree relatives with or without clinical symptoms were included in the present study.

The study has been approved by the Cyprus National Bioethics Committee (EEBK ΕΠ 2017.01.117). A written informed consent was obtained from the probands or their legal guardians to participate in this study.

### *CFTR* variant nomenclature and classification of CF‑causing mutations

For the *CFTR* variant nomenclature we used the recommendation of the Cystic Fibrosis Mutation Database (CFTR1) [4]. However, in the Discussion section we opted to use the more widely recognized ‘legacy nomenclature’ as indicated in parentheses. Variant pathogenicity was assessed according to the ‘Clinical and Functional Translation of CFTR’ database (CFTR2) [1] where applicable.

### Demographic and clinical data

Patients’ data included demographics, age and clinical presentation at diagnosis, sweat chloride concentrations (in mmol/L), *CFTR* genotype, and the most recent clinical data from both scheduled clinical visits and hospital admissions during the last 4.5 years, including clinical manifestations, anthropometrics, spirometry, airway microbiology, medical imaging, and treatment modalities. Patients’ age at follow up is generally reported as of July 31, 2024.

Clinical data were retrieved from patients’ medical records. Spirometry (Vitalograph Pneumotrac, Vitalograph Inc.; USA) was routinely performed in every clinical visit in patients above 6 years of age, according to the American Thoracic Society (ATS) and European Respiratory Society (ERS) guidelines [13]. The best FEV1 values per year are given in absolute values, in percent predicted and z-scores using the GLI Online Calculator® software system [14]. BMI is given in Kg/m^2^ for adult patients and in z-scores for children and adolescents using the WHO AnthroPlus® [15]. Sputum or cough swab microbiology testing was performed routinely in patients during each clinical follow-up. Chronic lung colonization with *Pseudomonas aeruginosa* was defined according to the modified Leeds criteria, i.e. when more than 50% of the cultures were positive for the pathogen, provided at least 4 cultures were performed in the previous year [16]. High-resolution chest computed tomography (HRCT) scans were performed in all symptomatic patients.

### Statistical methods

Categorical variables are presented as frequencies (%), while continuous variables are presented as mean (standard deviation). Categorical comparisons were calculated using the chi-squared test. All summary statistics and statistical comparisons were calculated using STATA 12 (Version 12, StataCorp, College Station; USA).

### Genetic Screening of the *CFTR* gene at diagnosis

Genotypic analysis of the *CFTR* gene was performed using the Devyser CFTR NGS kit (Devyser, Sweden) on an Illumina MiSeq platform (Illumina, San Diego, CA, USA) using the paired-end 300 cycles MiSeq Reagent Micro Kit v2 with a minimum coverage of 150X. Read alignment and variant calling were done with the Amplicon Suite software (SmartSeq, Novara, Italy).

### Genotyping with short tandem repeat (STR) genetic markers flanking the ***CFTR*** gene

**Fig. 1:**
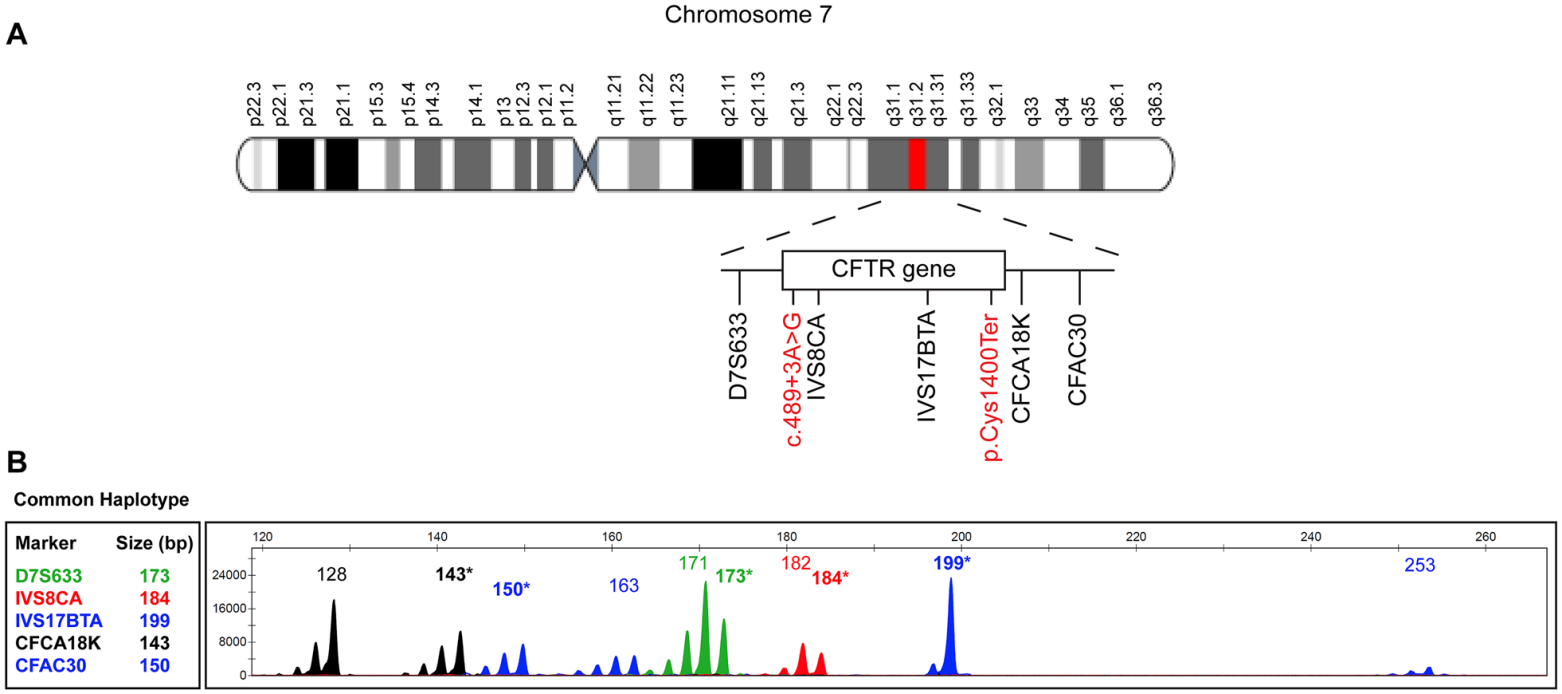
Herein are illustrated the five Short Tandem Repeat (STR) markers and their respective positions on chromosome 7, which were used to determine the common haplotype in individuals carrying the p.Cys1400Ter pathogenic variant. A. Five STR markers flanking the *CFTR* gene on chromosome 7 used for haplotype analysis. Red colour indicates the p.Cys1400Ter and c.489+3A>G positions relative to the STR markers. B. Representative electropherogram of fragment analysis of an individual with the p.Cys1400Ter carrying the common haplotype using the five STR markers. Asterisks indicate the sizes in base pairs found in the common haplotype

In the present study, five STR markers flanking the *CFTR* gene and according to *GRCh38.p14* genome assembly reference sequence: NC_000007.14 were used in a multiplex PCR and analyzed by capillary electrophoresis on a genetic analyzer [17–19]. These markers included D7S633, IVS8CA, IVS17BTA, CFCA18K and CFAC30. The reverse primer of each genetic marker was labelled with a fluorescent dye at 3′ (Fig. 1A) (Table 1). Multiplex PCR was performed using the QIAGEN multiplex PCR kit (QIAGEN) under the following conditions: 20 ng of genomic DNA, 12.5 μl QIAGEN Multiplex PCR Master Mix, 0.35 μM of each of IVS8CA primers, 0.3 μM of each of IVS17BTA primers, 0.2 μM of each of D7S633 primers, 0.4 μM of each of CFCA18K primers, 0.2 μM of each of CFAC30 primers and double distilled water to a final volume of 25 μl. The amplification reaction was initialized at 95 °C for 15 min, followed by 30 cycles for 40 s at 95 °C, 60 s at 58 °C; 60 s at 72 °C and a final extension for 5 min at 72 °C. Analysis of PCR products was performed by capillary electrophoresis on an ABI 3500xl Genetic Analyzer (Applied Biosystems) preceded by a denaturation step. A mix of 10 μl of HiDi™ formamide (Applied Biosystems) and 0.5 μl of GeneScan-600 LIZ size standard (Applied Biosystems) was added to 1 μl of diluted PCR product and denatured at 95 °C for 5 min. The mixture was immediately transferred on ice for 3 min and loaded for electrophoresis onto the ABI 3500xl genetic analyser (Applied Biosystems). PCR products were visualized and analyzed with GeneMapper™ v5 Software (Applied Biosystems). A representative example of a visualisation of a fragment analysis is illustrated in Fig. 1B.

**Table 1:** Primer sequences of STRs (Short Tandem Repeats) used in this study. Chromosome location observed fragment sizes and fluorescent labeling are indicated

STR Marker	Primer sequence (5’–3’)	Fluorescent Label	Chromosome location according to genome assembly *GRCh38.p14*	Observed sizes (bp)	Reference
D7S633	Fwd: TGAGCCTCGCATCACTG	–	117,370,884	163–181	[18]
	Rev: CTGGGGAGTCCTTTAACAGTA	VIC	117,370,752		
IVS8CA	Fwd: ACTAAGATATTTGCCCATTATCAAGT	–	117,548,291	180–196	[19]
	Rev: AATCTATCTCATGTTAATGCTGAAGA	PET	117,548,420		
IVS17BTA	Fwd: ATGCTGCATTCTATAGGTTATCAAT	–	117,611,892	199–257	[19]
	Rev: GACAATCTGTGTGCATCGG	6-FAM	117,612,098		
CFCA18K	Fwd: TATGAAGGAGACCAGGTCAAC	–	117,685,683	126–143	Current article
	Rev: CAAGGTCAGAAGTAGAGCCA	NED	117,685,782		
CFAC30	Fwd: AGAATAAAGCTTGGGAGTTGC	–	117,773,639	135–162	[17]
	Rev: CACTTCCTCTCCAACTTTGC	6-FAM	117,773,752		

## Results

Over the study period of 4.5 years, six non-related families of Cypriot descent were identified as harbouring the p.Cys1400Ter mutation. All subjects from these families that harboured the pathogenic mutation p.Cys1400Ter also exhibited co-segregation of the intronic variant c.489+3A>G within the same allele. We have not identified either the mutation p.Cys1400Ter or the variant c.489+3A>G in isolation in any alleles of CF patients or carriers that we have examined in Cyprus to date.

### CF cases bearing the *in cis* co-segregated combination of p.Cys1400Ter with c.489+3A>G

**Fig. 2:**
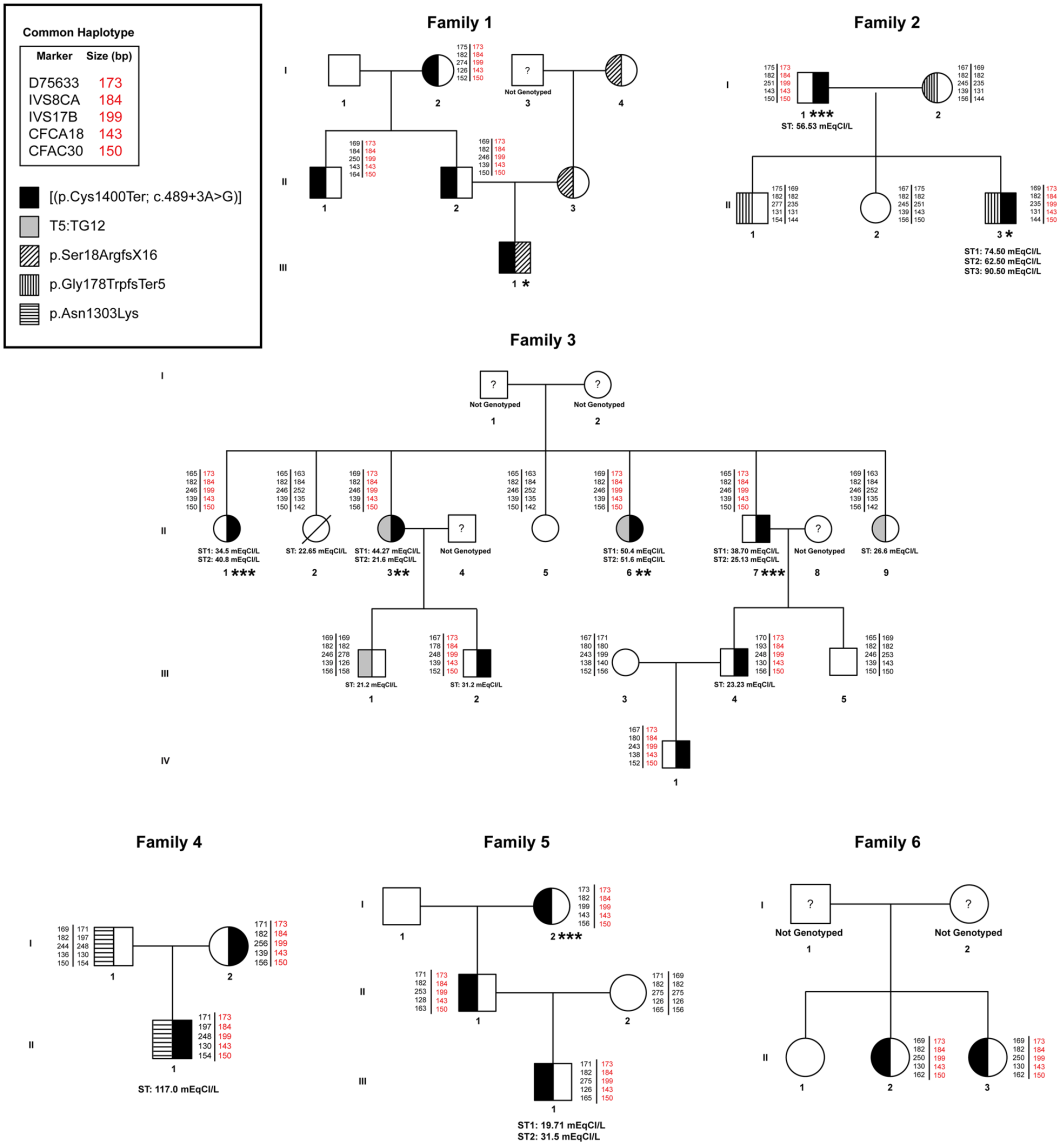
Pedigrees of six non-related families of Cypriot descent with individuals bearing alleles that harboured *in cis* c.489+3A>G and p.Cys1400Ter in the *CFTR* gene. Red colour indicates the common haplotype found in all individuals carrying the *in cis* c.489+3A>G and p.Cys1400Ter; ST: sweat test; * individuals with cystic fibrosis; ** individuals with CFTR-related disorder; *** individuals with possible CFTR-related disorder

Three individuals aged 5, 14 and 23 years old harboured p.Cys1400Ter with c.489+3A>G *in cis,* in compound heterozygosity with another known *CFTR* pathogenic mutation (p.Ser18ArgfsTer16, p.Gly178TrpfsTer5 and p.Asn1303Lys, respectively) (Table 2). All three cases were diagnosed in the first year of life with persistent respiratory symptoms, had elevated sweat chloride (> 60 mmol/L) and typical clinical phenotype with pancreatic insufficiency. The two older individuals have reduced FEV1 z-scores at − 2.6 (1.71 L, 68% predicted) and − 2.7 (3.19 L, 68% predicted), as well as extensive bronchiectatic changes. One of them has been recently commenced on CFTR modulator (elexacaftor, tezacaftor, ivacaftor) because of the other *CFTR* mutation he bears [p.Asn1303Lys] [20] with good clinical response (Table 2, Fig. 2).

**Table 2:** Diagnostic and clinical features of three CF, two CFTR-RD and four possible CFTR-RD cases identified with the *in cis* c.489+3A>G variant and p.Cys1400Ter pathogenic mutation

Diagnosis	CF Cases			CFTR-RD Cases		Possible CFTR-RD Cases			
Families/Cases	Family 1 Case 1	Family 2 Case 1	Family 3 Case 1	Family 4 Case 1	Family 4 Case 2	Family 2 Case 2	Family 4 Case 3	Family 4 Case 4	Family 5 Case 1
Allele 1 mutation(s)	p.Cys1400 Ter/c.489+3 A>G	p.Cys1400 Ter/c.489+3A> G	p.Cys1400Ter/c.489+3A> G	p.Cys1400 Ter/c.489+3 A>G	p.Cys1400 Ter/c.489+3 A>G	p.Cys1400Ter/c.489+3 A>G	p.Cys1400 Ter/c.489+3 A>G	p.Cys1400 Ter/c.489+3 A>G	p.Cys1400 Ter/c.489+3 A>G
Allele 2 mutation(s)	p.Ser18 ArgfsTer16	p.Gly178 TrpfsTer5	p.Asn1303Lys/c.3469-65C > A	cDNA. 5T:TG12	cDNA. 5T:TG12	None	None	None	None
Gender	Female	Male	Male	Female	Female	Male	Female	Male	Female
Age (*years*)	5.9	14	23.3	69.9	66.2	61.6	75.2	64.6	69.6
Age at diagnosis (*years*)	0.74	0.9	0.4	66	63	60	71	61	67
Sweat chloride (*mmol/L*)^1^	93	82.5	117	32.9	51	56.5	37.6	31.9	ND
Clinical presentation	Neonatal screening	Persistent respiratory symptoms; Electrolyte imbalance	Persistent respiratory symptoms; Electrolyte imbalance; Failure to thrive	Persistent respiratory symptoms	Persistent respiratory symptoms	Persistent respiratory symptoms	Persistent respiratory symptoms	Persistent respiratory symptoms	Persistent respiratory symptoms
Digital clubbing	No	Yes	Yes	No	No	No	No	No	No
BMI z-score (absolute value)^2^	− 1.0 (14)	− 0.2 (18.3)	0.7 (25.7)	0.2 (22.3)	− 1.5 (18.2)	1.7 (31.5)	− 0.6 (20.1)	0.1 (23.6)	1.1 (27)
FEV_1_ z-score (absolute value, % predicted)^3^	ND	− 2.6 (1.71 L, 68%)	− 2.7 (3.19 L, 68%)	− 2.3 (1.31 L, 63%)	− 3.2 (1.15 L, 50%)	− 0.4 (3.07 L, 94%)	− 1.7 (1.10 L, 68%)	− 4.5 (0.75 L, 26%)	− 0.9 (2.22 L, 86%)
Pancreatic insufficiency^4^	Yes	Yes	Yes	No	No	No	No	No	No
Chronic colonization^5^	No	Pseudomonas aeruginosa	Staphylococcus aureus	No	No	No	No	No	No
Chest CT abnormal findings (BE, ATL, EMPH)^6^	RUL: No LUL: No RML: No LING: No RLL: No LLL: No	RUL: BE LUL: BE RML: BE, ATL LING: BE RLL: BE LLL: BE	RUL: BE LUL: BE RML: BE LING: BE RLL: BE LLL: BE	RUL: BE LUL: BE RML: BE LING: BE RLL: BE LLL: BE	RUL: BE LUL: BE RML: BE, ATL LING: BE, ATL RLL: BE LLL: BE	RUL: EMPH LUL: EMPH RML: EMPH LING: EMPH RLL: EMPH LLL: EMPH	RUL: BE LUL: BE RML: BE LING: BE RLL: BE, ATL LLL: BE, ATL	RUL: EMPH LUL: EMPH RML: EMPH LING: BE, EMPH RLL: EMPH LLL: BE, EMPH	RUL: No LUL: No RML: BE LING: No RLL: BE LLL: BE
Intake of CFTR modulators	No	No	Yes	No	No	No	No	No	No

^1^Mean value (mmol/L) of all performed sweat chloride tests; ^2^BMI z-score and absolute value measured on date of best FEV_1_ measurement in follow-up year; ^3^Best FEV_1_ z-score, absolute value and percent predicted measured in follow-up year; ^4^Pancreatic insufficiency defined as pancreatic enzyme supplementation requirement; ^5^Chronic pulmonary colonization with *Pseudomonas aeruginosa* defined according to modified Leeds criteria; ^6^Abnormal findings revealed by chest CT scan (BE: Bronchiectasis, ATL: Atelectasis, EMPH: Emphysema); RUL: Right upper lobe; LUL: Left upper lobe; RML: Right middle lobe; LING: Lingula; RLL: Right lower lobe; LLL: Left lower lobe; CFTR-RD: CFTR-related disease; ND: not done

### CFTR-RD cases bearing the *in cis* co-segregated combination of p.Cys1400Ter with c.489+3A>G

Two female siblings aged 69 and 66 years carrying *in cis* the p.Cys1400Ter with c.489+3A>G were found to be in compound heterozygosity with cDNA 5T:TG12, which is known to have variable functional effect and controversial clinical consequences [21] (Fig. 2). The first patient was diagnosed at the age 66 years with an average sweat chloride of 33 mmol/L (sweat test 1: 44.3 mmol/L, sweat test 2: 21.6 mmol/L). She had a BMI of 22.3 with no clinically overt manifestations of pancreatic insufficiency and established features of chronic lung disease for years with reduced FEV1 z-score at − 2.3 (1.31 L, 63.2% predicted) and bronchiectasis. On chest CT, she had moderate cylindrical bronchiectasis at both upper lobes and severe bronchiectatic changes at the middle and lower lobes bilaterally (Table 2). The second patient was diagnosed at the age of 63 years, after identification of her sister, and had an average sweat chloride of 51 mmol/L (sweat test 1: 40.8 mmol/L, sweat test 2: 34.5 mmol/L). Although her BMI was at 18.2, she had no clinically overt manifestations of pancreatic insufficiency and established features of chronic lung disease for years with reduced FEV1 z-score at − 3.2 (1.15 L, 50.2% predicted) and bronchiectasis. On chest CT, she had severe bronchiectatic changes and subsegmental atelectasis at the right middle lobe and the lingula and mild bronchiectatic changes at both upper lobes and both lower lobes (Table 2).

### Possible CFTR-RD cases bearing the *in cis* co-segregated combination of p.Cys1400Ter with c.489+3A>G

Four individuals aged between 61 and 75 years had a phenotype of possible CFTR-RD and were found to harbour *in cis* p.Cys1400Ter with c.489+3 A> G but no variant on the other allele on *CFTR* sequencing. All presented for diagnostic work-up at advanced age (between 60 to 71 years of age) and the sweat tests performed in three of them yielded equivocal results (sweat chloride 31.9, 37.6 and 56.5 mmol/L). They had manifestations of chronic lung disease for many years, reduced FEV1 z-scores ranging from − 0.4 (3.07 L, 94.5% predicted) to − 4.5 (0.75 L, 25.9% predicted) in three of them, bronchiectasis and/or emphysema on chest CTs. Although fecal elastase was not assessed in these patients, they had no clinically overt pancreatic insufficiency with no manifestations of malabsorption and BMI ranging from 31.5 to 20.1 (Table 2, Fig. 2).

### Carriers bearing the *in cis* co-segregated combination of p.Cys1400Ter with c.489+3A>G

Eleven individuals (median age: 35.2, range: 2.9; 67.2 years) were found to harbour p.Cys1400Ter with c.489+3A>G *in cis*, had no variant on the other allele on CFTR sequencing and had not displayed any suspect clinical manifestations for CF up to the end of the study period.

### Haplotype analysis of families bearing the *CFTR* p.Cys1400Ter; c.489+3A>G combination

Haplotype examination using five STR markers flanking the *CFTR* gene was performed by fragment analysis in 20 subjects carrying the p.Cys1400Ter in co-segregation with the intronic variant c.489+3A>G on the same allele. The subjects were three patients with CF, two with CFTR-RD, four with possible CFTR-RD (Table 2) and 11 carriers. Additionally, nine family members who did not carry the p.Cys1400Ter in co-segregation with the intronic variant c.489+3A>G on the same allele and 20 random samples, which were selected from the DNA bank of the MGFT department at the Cyprus Institute of Neurology and Genetics, also underwent the same haplotype analysis. Haplotype analysis revealed a specific common haplotype in all individuals who carried the complex mutation *in cis,* suggesting that they originated from a common ancestor (Fig. 1B).

## Discussion

This study provides evidence that the c.489+3A>G variant co-segregates in Cyprus with p.Cys1400Ter as an ancestral variants combination that has spread on the island due to a possible founder effect. Based on the findings of this study, further analysis should be conducted to determine whether co-segregation is present in other Mediterranean countries. Special attention should be given to the findings from isolated case reports involving patients of Algerian ancestry, as documented in CFTR-France [8, 9]. Conducting further analysis to determine whether co-segregation exists in other Mediterranean countries is important for several reasons. First, it could provide valuable insights into the geographic distribution and historical spread of these variants, potentially confirming a shared ancestral origin or founder effect in the region. Second, understanding the prevalence and patterns of these mutations across different populations could help refine diagnostic and screening strategies, particularly in populations with shared genetic backgrounds. Finally, integrating data from isolated case reports into broader analyses would enhance our understanding of the clinical spectrum and variability of these mutations, ultimately improving patient management and genetic counselling in affected communities.

The clinical utility of testing for co-segregation largely depends on whether it contributes to understanding disease mechanisms, impacts phenotype or informs clinical management. Currently, no direct evidence from the current study suggests that co-segregation of p.Cys1400Ter and c.489+3A>G affects the CF phenotype or disease severity in cases with an additional CF-causing mutation. Without such evidence, routine screening for co-segregation in broader populations may have limited clinical relevance, especially when another pathogenic *CFTR* mutation explains the clinical phenotype.

Two of our patients bearing the *in cis* co-segregated combination of p.Cys1400Ter with c.489+3A>G displayed a compound heterozygosity with the Poly-T tract and the TG tract 5T:TG12 and developed CFTR-RD and chronic lung disease with ascending age, characterised by reduced lung function, bronchiectasis and equivocal sweat test results. The 5T:TG12 tract has been previously found to produce low levels of correctly spliced CFTR mRNA. The high variability and low levels of residual functionality of the 5T:TG12 haplotype has been reported to cause CFTR-RD, but also mild forms of CF [21]. More specifically, the 5T:TG12 tract undergoes a high anomalous splicing producing a low quantity of wild-type CFTR mRNA, as confirmed in CF patients with pancreatic sufficiency, CFTR-RD and congenital bilateral absence of the vas deferens. The distribution of bronchiectasis on chest CT can point towards a specific aetiology, with upper lobe predominant disease found in CF and lower and middle lobe predominant disease in non-CF conditions [11]. However, bronchiectasis in cystic fibrosis is usually widespread, and although upper lobe involvement is almost universal, both central and peripheral bronchiectasis is present in approximately two thirds of patients [22–24]. We believe that in the absence of functional assessments, such as the nasal potential difference or intestinal current measurements, we cannot claim that these two patients have mild CF [11].

Interestingly, four of the 15 (27%) individuals in our study harboured the *in cis* co-segregated combination of p.Cys1400Ter and c.489+3A>G with no other *CFTR* allele variant. Despite this, they developed respiratory symptoms and abnormalities on CT chest imaging, bronchiectasis and/or emphysema, at advanced age (60 and 71 years). Unfortunately, electrophysiological analysis of CFTR protein function for these individuals was not carried out due to lack of availability of nasal or intestinal epithelia potential difference. It is therefore possible that these patients may have a diagnosis of CFTR-RD, but for the purposes of this study they were considered to have ‘‘possible’’ CFTR-RD [24].

Of interest are also the asymptomatic 11 individuals who were characterized as carriers. They are quite younger (median age 35.2 years) than the symptomatic individuals with possible CFTR-RD and in light of the development of chronic lung disease in the older patients with the same CFTR genotype, we cannot exclude in some of them the possibility to develop chronic lung disease with ascending age. Careful follow-up of these individuals is warranted to detect and manage accordingly the appearance of any respiratory manifestations in the future [25].

In conclusion, this study provides evidence that the c.489+3A>G variant co-segregates with p.Cys1400Ter, suggesting that they may represent an ancestral variant combination in Cyprus, possibly due to a founder effect. Additionally, this combination may also be prevalent in other Mediterranean countries. In the CFTR2 database, c.489+3A>G is currently classified as a variant of uncertain significance, while p.Cys1400Ter is recognized as a rare and severe pathogenic variant. It is essential to perform a comprehensive analysis of the *CFTR* gene, including the assessment of gene rearrangements, before providing genetic counselling to individuals identified through population screening as carriers of this variant. Accurate identification is critical for the correct clinical diagnosis and management of the disease.

The co-segregation of p.Cys1400Ter with c.489+3A>G, even in the absence of additional pathogenic variants on the other *CFTR* allele, may be associated with chronic lung disease or potential CFTR-related disorders (CFTR-RD) in older individuals. Further research is necessary with the performance of vitro or in vivo functional studies of the c.489+3A > G variant and its interaction with p.Cys1400Ter to fully elucidate their clinical implications.

## Data Availability

The datasets used and analysed during the current study are available from the corresponding author on reasonable request.

## Abbreviations

CFTR: Cystic fibrosis transmembrane regulator
PwCF: People with cystic fibrosis
CFTR-RD: CFTR-related disorders
STR: Short tandem repeat
